# ﻿Seasonal and microclimatic effects on leaf beetles (Coleoptera, Chrysomelidae) in a tropical forest fragment in northeastern Mexico

**DOI:** 10.3897/zookeys.1080.76522

**Published:** 2022-01-04

**Authors:** José Norberto Lucio-García, Uriel Jeshua Sánchez-Reyes, Jorge Víctor Horta-Vega, Jesús Lumar Reyes-Muñoz, Shawn M. Clark, Santiago Niño-Maldonado

**Affiliations:** 1 Tecnológico Nacional de México-Instituto Tecnológico de Cd. Victoria, Blvd. Emilio Portes Gil No. 1301, C.P. 87010. Cd. Victoria, Tamaulipas, México Tecnológico Nacional de México-Instituto Tecnológico de Cd. Victoria Victoria Mexico; 2 Facultad de Ciencias Biológicas, Universidad Juárez del Estado de Durango. Av. Universidad S/N, Fracc. Filadelfia, 35010 Gómez Palacio, Durango, Mexico Universidad Juárez del Estado de Durango Durango Mexico; 3 Brigham Young University, Life Science Museum, Provo, Utah 84602, USA Brigham Young University Provo United States of America; 4 Universidad Autónoma de Tamaulipas, Facultad de Ingeniería y Ciencias, Centro Universitario Victoria, C.P. 87149. Cd. Victoria, Tamaulipas, México Universidad Autónoma de Tamaulipas Victoria Mexico

**Keywords:** Abiotic factors, community response, ecological niche, phytophagous insects, seasonal changes

## Abstract

Leaf beetles (Coleoptera: Chrysomelidae) constitute a family of abundant, diverse, and ecologically important herbivorous insects, due to their high specificity with host plants, a close association with vegetation and a great sensitivity to microclimatic variation (factors that are modified gradually during the rainy and dry seasons). Therefore, the effects of seasonality (rainy and dry seasons) and microclimate on the community attributes of chrysomelids were evaluated in a semideciduous tropical forest fragment of northeastern Mexico. Monthly sampling was conducted, between March 2016 and February 2017, with an entomological sweep net in 18 plots of 20 × 20 m, randomly distributed from 320 to 480 m a.s.l. Seven microclimatic variables were simultaneously recorded during each of the samplings, using a portable weather station. In total, 216 samples were collected at the end of the study, of which 2,103 specimens, six subfamilies, 46 genera, and 71 species were obtained. The subfamily Galerucinae had the highest number of specimens and species in the study area, followed by Cassidinae. Seasonality caused significant changes in the abundance and number of leaf beetle species: highest richness was recorded in the rainy season, with 60 species, while the highest diversity (lowest dominance and highest H’ index) was obtained in the dry season. Seasonal inventory completeness of leaf beetles approached (rainy season) or was higher (dry season) than 70%, while the faunistic similarity between seasons was 0.63%. The outlying mean index was significant in both seasons; of the seven microclimatic variables analyzed, only temperature, heat index, evapotranspiration and wind speed were significantly related to changes in abundance of Chrysomelidae. Association between microclimate and leaf beetles was higher in the dry season, with a difference in the value of importance of the abiotic variables. The results indicated that each species exhibited a different response pattern to the microclimate, depending on the season, which suggests that the species may exhibit modifications in their niche requirements according to abiotic conditions. However, the investigations must be replicated in other regions, in order to obtain a better characterization of the seasonal and microclimatic influence on the family Chrysomelidae.

## ﻿Introduction

Accelerated loss of biological diversity, as well as the alterations in native ecosystems as a result of human activities, are among the most important environmental issues at a global level (Challenger and Dirzo 2009). These include land cover fragmentation, overexploitation of natural resources, pollution, and climate change (Hautier et al. 2015).

Abiotic modification produces direct effects on organisms, affecting physiology, behavior, and reproduction (Uribe-Botero 2015). Changes in precipitation and increased environmental temperature (Schaefer et al. 2008) are likely to cause alterations in abundance and even loss of species (Brook et al. 2008), as well as changes in their geographical distribution (Parmesan and Yohe 2003; Root et al. 2003). However, these responses are variable, based on the type of organism and its niche breadth (Vié et al. 2009). Therefore, changes in climatic abiotic variables are key factors in the composition and structure of biological communities, besides other ecological aspects (Pimm 2007), such as seasonal changes during wet and dry seasons (Wolda 1988; Rzedowski 2006).

An aspect of greatest influence on these communities is the microclimate (Cloudsley-Thompson 1962). This is the result of local spatial and seasonal variations in climate and has been shown to play an important role in the dynamics of metapopulations (Checa et al. 2014). Likewise, it is essential for the survival and development of the species, affecting larval diapause or growth, or indirectly modifying the availability of food resources (Currano et al. 2008; DeLucia et al. 2008). The microclimate is related to seasonal variations in the communities of phytophagous insects (Chen et al. 1999), but its specific influence has been scarcely studied.

Phytophagous insects are among the most important trophic groups that respond significantly to climatic changes. Their presence is key in natural or anthropic ecosystems, either playing a relevant role in nutrient cycling processes, or in the diet of other organisms (Iannacone and Alvariño 2006). Furthermore, their physiological processes are determined by the conditions of the environment (Régnière 2009).

Leaf beetles (Coleoptera: Chrysomelidae) constitute a model family to evaluate the seasonal effects of abiotic variation on herbivorous insect communities, since they occupy one of the first places in worldwide diversity (Santiago-Blay 1994). Most chrysomelid species exhibit phytophagous feeding habits and a close relationship with their host plants, as well as a great sensitivity to microclimatic variation (Niño-Maldonado and Sánchez-Reyes 2017). Also, they are considered to be a group with important potential for monitoring natural areas (Furth et al. 2003).

The present study was carried out in a semideciduous tropical forest (STF) fragment in the municipality of Victoria, Tamaulipas, in northeastern Mexico. The area is included in the biogeographic province of the Sierra Madre Oriental and is located within one of the 15 panbiogeographic nodes of Mexico (Morrone and Márquez 2008). Therefore, it constitutes a region with a high priority for conservation (CONABIO et al. 2007). Despite this, there are no studies in STF evaluating the effect of seasonality and microclimate on the family Chrysomelidae. It is important to recognize the factors that restrict the distribution of the species, and thus further delimit efficient conservation strategies of this important area. Based on the above, the objectives of this study were 1) to prepare a faunistic list of chrysomelid species, 2) to compare their richness, abundance, and diversity between seasons, 3) to define the abiotic variables seasonally related to the presence and abundance of the species, and 4) to delimit the breadth niche and categorize the leaf beetles as specialists or generalists, based on their variation related to the seasonal abiotic environment.

## ﻿Materials and methods

### ﻿Study area

**Figure 1.**
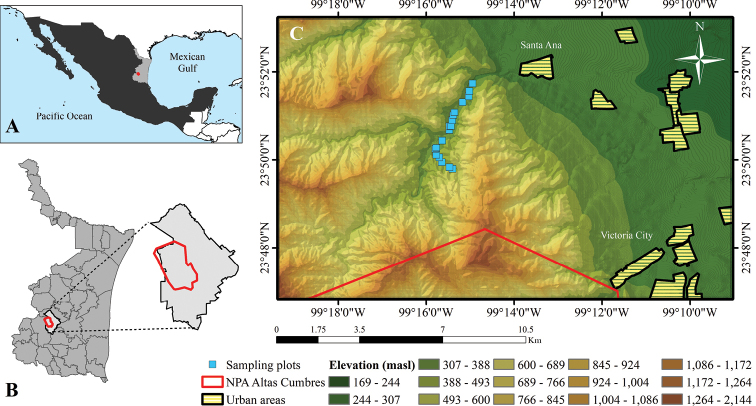
Location of the study area. **A** Ejido Santa Ana (red point) in Tamaulipas State, Mexico **B**NPA Altas Cumbres (red polygon) within Victoria municipality in Tamaulipas **C** Distribution of the sampling plots (blue squares) in the semideciduous tropical forest.

The study area of semideciduous tropical forest (**STF**) is located in the Ejido Santa Ana, municipality of Victoria, in the center of the state of Tamaulipas, northeastern Mexico 23°52'4.27"N, 99°13'51.37"W and 23° 47'23.06"N, 99°18'10.22"W (DMS) (Fig. 1). It is included in the biogeographic province of the Sierra Madre Oriental (**SMO**), converging to the south with Peregrina Canyon, within the Natural Protected Area (**NPA**) “Altas Cumbres.”

Two climate groups characteristic of Tamaulipas were observed in the area: 1) Semi-warm, sub-humid, with summer rains, averaging temperatures between 16.4 °C and 29.2 °C, and 2) Semi-warm, semi-dry subtype, with average temperatures from 15.1 °C to 22.9 °C. The average annual precipitation is 577 mm, with May to October as the wettest months (rainy season) and November to April having the lowest precipitation (dry season) (Gobierno del Estado 2015). Regarding the semideciduous tropical forest, it is the second richest ecosystem in plant species of the state of Tamaulipas and is located between 350 and 500 m a.s.l, comprising areas adjacent to the margin of rivers and streams. Therefore, this habitat conserves higher environmental humidity for most of the year, protecting it from sudden climatic changes, such as sudden temperature differences (García-Morales et al. 2014).

### ﻿Sampling

A total of 18 plots measuring 20 × 20 m (400 m^2^) was randomly established over an approximate land area of 5 km^2^. Plots were distributed in areas of dense herbaceous and shrub vegetation, separated at least by 10 meters from the main road, in order to minimize anthropogenic influence. Each plot was measured and delimited with a 50 m tape, using trunks, trees, or branches as vertices; the center of the plot was georeferenced with a Garmin Etrex 30 GPS and then marked with a brightly colored ribbon to facilitate its location in the field.

Beetles were sampled with an entomological sweep net of 60 cm length and 40 cm rim diameter. In each plot (sample unit), 200 net beats were made, covering all the sampling area zigzagging on the understory vegetation. The contents of the net were placed inside a polyethylene bag with 70% alcohol and a collecting label data. All of the 18 plots were sampled from 10:00 to 17:00 hours, once a month, from March 2016 to February 2017.

Sample bags were processed in the Entomology Laboratory of the Facultad de Ingeniería y Ciencias, Universidad Autónoma de Tamaulipas. Each sample was placed in a tray with water, plant debris were then removed using entomological forceps, and the insect specimens were afterwards placed in small bottles with 70% alcohol. Later, the contents of each bottle were analyzed in a Petri dish, using a stereoscopic microscope to identify the specimens; chrysomelids were dried on absorbent paper and mounted in opaline triangles, following the methodology of Triplehorn and Johnson (2005). Taxonomic determination of subfamilies was carried out using the keys of Triplehorn and Johnson (2005), while genera and/or species were identified by consulting various authors (Wilcox 1972; Scherer 1983; White 1993; Flowers 1996; Riley et al. 2002; Staines 2002), as well as by comparison with previously identified specimens.

Microclimatic variables were recorded using a Kestrel 3500 portable meteorological station, with which the following variables were evaluated: maximum wind speed (m/s), average wind speed (m/s), temperature (°C), relative humidity (%), heat index (°C), dew point (°C) and evapotranspiration (°C). Abiotic data collection was carried out in each plot, simultaneously with the sampling of leaf beetles (once a month for each plot, during the period from March 2016 to February 2017).

### ﻿Data analysis

Statistical differences in abundance and number of species between seasons were calculated with a non-parametric Mann-Whitney test and a diversity permutation test, respectively. Both analyses were conducted using PAST 3.17 software (Hammer et al. 2017).

Seasonal estimated richness was determined using Chao 1, Chao 2, Jackknife 1 and ACE non-parametric estimators. These indices are recommended for the minimum estimate of richness and useful as a complementary measure in biodiversity analyzes (Gotelli and Colwell 2011). Chao 1 considers the abundance of rare species (singletons and doubletons). Chao 2 is robust for presence-absence data. Jackknife 1 is a conservative index based on incidence data of those species found only in a single sample, while ACE is an index that considers the abundance of species represented by 1–10 individuals (Magurran 2004). The estimators were calculated by means of 100 randomizations without replacement in the software EstimateS 9.1.0 (Colwell 2013), based on the abundance of the recorded species. In addition, the Clench model was used to calculate the estimated species richness, following the methods proposed by Jiménez-Valverde and Hortal (2003). This procedure was performed in STATISTICA 8.0 (StatSoft, Inc. 2007).

Alpha diversity was estimated using Shannon’s entropy index (**H**’) and Simpson’s dominance index (**D**). Both values were transformed to the effective number of species (true diversity), through the Hill numbers of order (**q**) 1 and 2, respectively (Jost 2006). To measure beta diversity, the Bray-Curtis similarity index was used, which relates the abundance of the shared species with the total abundance in two samples. Therefore, it constitutes a robust measure for the analysis of biotic similarity between communities (Magurran 2004). All diversity analyses were carried out with PAST software.

Association between leaf beetle species and the environmental abiotic variables, as well as the measure of niche breadth, were calculated with the Outlying Mean Index (**OMI**). This index identifies the niche of the species, or marginality, according to the average distance between the abiotic resources used by each species (centroid) with respect to the total resources available (microclimate) in the area. It gives a more even weight to all sampling units, including those with a low number of species or individuals (Dolédec et al. 2000). First, the OMI assesses the contribution of the abiotic variables to the niche separation of the species by computing a Principal Component Analysis, and higher correlation values (loadings) are interpreted at each of the most important axes. Then, a total Inertia (**InerO**) value is obtained, which is a measure proportional to the average marginality of the species and represents a quantification of the influence of environmental variables on the separation of the species niche. Lastly, the analysis decomposes the inertia associated with the distribution of a species (**InerO**) into three main parameters: Marginality, Tolerance (**T1**), and Residual Tolerance (**T2**) (Dolédec et al. 2000).

Marginality represents the deviation of the environmental conditions used by a species with respect to the average environment for the entire study area. Species with high OMI values ​​have marginal niches (occur in atypical habitats, and are influenced by a specific subset of environment variables), while those with low values ​​have non-marginal niches (common species occurring in typical habitats, without a specific response to environment variables). Tolerance (T1) measures the dispersion of the assessment units that contain a species along an environmental gradient (the range of habitat of the species), and it is analogous to the concept of niche breadth: high tolerance values ​​represent greater niche breadth, and the species are distributed in habitats with widely variable conditions (generalist); contrarily, low tolerance values ​​indicate a smaller niche width where a species is distributed in habitats with a limited range of conditions (specialists). Finally, T2 is defined as the variance in the species niche that is not considered by the marginality axes, and it is useful for determining the reliability of a set of environmental conditions for the definition of the niche of each species (Dolédec et al. 2000).

Statistical significance of the OMI was determined with a Monte Carlo test, in which the observed marginalities are compared with 10,000 random permutations, in order to reject the null hypothesis that species are equally distributed in relation to (not influenced by) environmental variables (Dolédec et al. 2000). All OMI analyses were carried out in ADE-4 software (Thioulouse et al. 1997), and they were calculated separately for the rainy and dry seasons. Data input consisted of a matrix with the abundances of each of the species in each month/season and a matrix with the values ​​of the seven environmental variables registered in each of the sampling plots. Ordination graphics of centroids and loadings were generated in the same software and later exported to CorelDRAW X3 to be edited. Environmental ranges of species were calculated for each of the significant variables using the Kriging interpolation technique, which is a geostatistical method that quantifies spatial autocorrelation for the prediction and generation of continuous surfaces (Murillo et al. 2012). Procedures were carried out in ArcGis 10.2.2 (ESRI 2014).

## ﻿Results

### ﻿Overall response of leaf beetles in the semideciduous tropical forest

During the study, 2,103 specimens of Chrysomelidae were obtained, involving six subfamilies, 47 genera and 71 species (Appendix 1: Table A1). Galerucinae were most abundant (1,628 specimens = 77%), followed by Cassidinae (410 = 19.44%). Among the other four subfamilies, only 65 specimens (3%) were collected throughout the year, being 36 in Eumolpinae, 14 in Criocerinae, nine in Chrysomelinae, and six in Cryptocephalinae. Regarding total richness, Galerucinae represented 51% (36 species), Cassidinae 17% (12 species), Eumolpinae 11% (eight species), Chrysomelinae 8% (six species), Criocerinae 7% (five species), and Cryptocephalinae 6% (four species).

Species that dominated in abundance in the study area were *Centralaphthonadiversa* (Baly, 1877) (629 individuals), *Monomacrabumeliae* (Schaeffer, 1905) (528 individuals), *Heterispavinula* (Erichson, 1847) (311 individuals), and *Margaridisa* sp. 1 (147 individuals), which together represent 77% (1,615 individuals) of the total abundance recorded. In addition, the community included 67 species with very low abundances, from which 25 (37%) correspond to singletons and nine to doubletons (13%). The dominance value (D) in the study area was 0.1998, which represents a true diversity (1/D) of 5.005. For the Shannon index (H’), a value of 2.221 was registered, with true diversity (*e*^H^) of 9.217.

### ﻿Seasonal variation

Seasonal differences in abundance of the leaf beetle community were statistically significant (Mann-Whitney U = 4039; p ≤ 0.0001). The highest number of specimens was recorded during the rainy season (1,242 specimens, involving 41 genera), followed by the dry season (861, involving 30 genera). According to the permutation test, significant differences were also found in the number of species and diversity. Highest species richness was recorded in the rainy season. In contrast, the lowest dominance and highest diversity were obtained in the dry season (Table 1).

**Table 1.** Diversity permutation test for species richness and alpha diversity of leaf beetles between seasons.

	Season		
	Rainy	Dry	p
Observed species richness	60	40	0.0132
Simpson index (D)	0.228	0.175	0.0001
Shannon index (H´)	2.062	2.232	0.0229

Estimated species richness according to the non-parametrical estimators in the rainy season ranged between 85 and 100 species; therefore, the observed richness represents between 59.66 and 69.96% of completeness. For the dry season, the estimated richness varied from 48 to 56 species, indicating a completeness from 70.49 to 82.85% (Table 2). Inventory reliability with Clench’s model was higher during the dry season, with a completeness of 81% and a lower slope value, compared with the rainy season (Table 2).

**Table 2.** Chrysomelid estimated species richness and sampling completeness during the rainy and dry seasons.

Estimator	Rainy	% of completeness	Dry	% of completeness
Chao 1	97.53	61.52	55.11	72.58
Chao 2	90.44	66.34	56.74	70.49
Jack 1	85.76	69.96	55.88	75.64
Ace	100.56	59.66	48.28	82.85
Clench model (slope)	0.1561	–	0.077	–
Clench model (estimated richness)	82	73	50	81

% was obtained on the basis of observed species richness.

The best represented subfamily during the rainy season was Galerucinae (943 specimens, 32 species), followed by Cassidinae (260, 11 species). This same pattern was reflected in the dry season: Galerucinae with 685 specimens (21 species), followed by Cassidinae with 150 specimens (7 species). The remainder of the subfamilies had lower abundances and number of species for both seasons (Table 3).

**Table 3.** Number of specimens and species registered by subfamily and season in the semideciduous tropical forest.

	Season			
	Rainy		Dry	
Subfamily	Specimens	Species	Specimens	Species
Galerucinae	943	32	685	21
Cassidinae	260	11	150	7
Eumolpinae	25	7	11	5
Criocerinae	7	3	7	2
Chrysomelinae	5	5	4	3
Cryptocephalinae	2	2	4	2

Faunistic similarity according to the Bray-Curtis index was 0.63%. A high proportion of the species composition shared between seasons involved Galerucinae, including *Acrocyumdorsale* Jacoby, 1885, *C.diversa*, *Epitrix* sp. 1, *Margaridisa* sp. 1, and *Monomacrabumeliae*. The proportion was also high for Cassidinae, involving *Brachycorynapumila* Guérin-Méneville, 1844, *Helocassiscrucipennis* (Boheman, 1855), and *Heterispavinula* (Erichson, 1847).

### ﻿Response of Chrysomelidae to seasonal microclimatic variation

**Figure 2.**
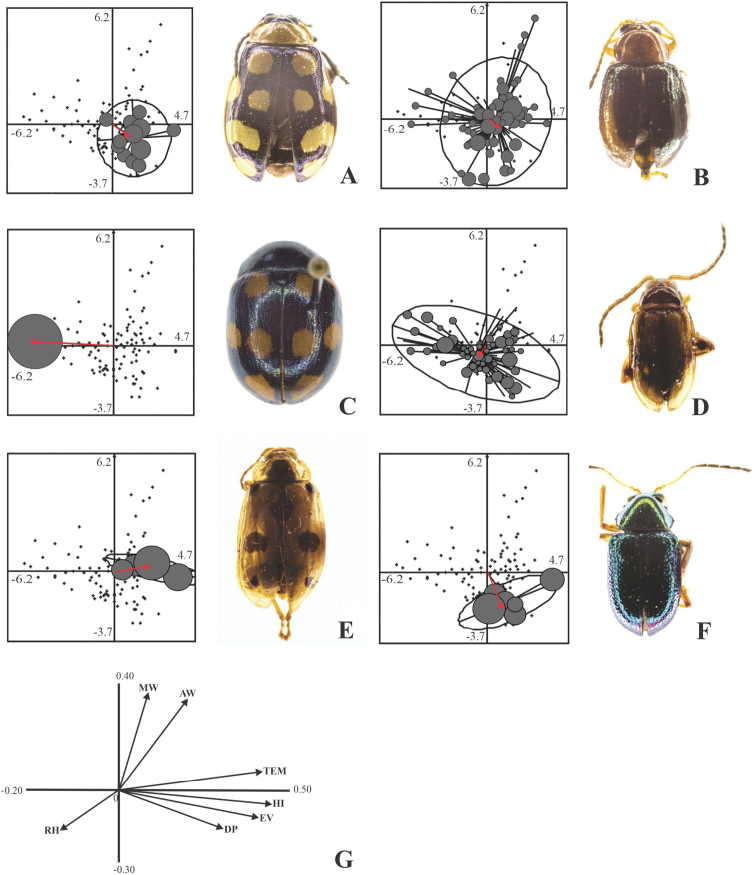
Individual dispersion of leaf beetle species whose association for microclimatic variables was significant in the rainy season **A***Alagoasatrifasciata***B***Centralaphthonadiversa***C***Labidomerasuturella***D***Monomacrabumeliae***E***Walterianella* sp. 1 **F***Zenocolaspisinconstans*. At each species panel: the gray circles represent the presence of the species in the sample, and the size of the circle is proportional to its abundance; straight lines represent vectors and indicate the dispersion of the species from the average position (centroid) towards each of the evaluation units where it was recorded; and ellipses represent the concentration of 95% of the specimens of the species. **G** canonical correlation values ​​(loadings) between microclimatic variables and the abundance of Chrysomelidae. Abbreviations: MW: Maximum wind speed, AW: average wind speed, Tem: temperature, RH: relative humidity, HI: heat index, DP: dew point, Ev: evapotranspiration.

The OMI analysis for the rainy season indicated a significant deviation between the abiotic conditions used by the leaf beetles and the average total microclimatic conditions (Monte Carlo test, *p* = 0.047). Of the 60 species registered in this season, only six showed a significant association. *Centralaphthonadiversa* and *M.bumeliae* obtained low marginality values, which represents a wider niche breadth, and they were thus considered to be generalist species (Table 4); abundance of these species was equally distributed in almost all samples (Fig. 2). The rest of the species presented high marginality ​​and lower tolerance values, which indicates a smaller niche breadth, and they were therefore categorized as specialists. *Labidomerasuturella* Guérin-Méneville, 1838 was the species with the highest marginality and the lowest tolerance, followed by *Walterianella* sp. 1, *Zenocolaspisinconstans* (Lefèvre, 1878) and *Alagoasatrifasciata* (Fabricius, 1801) (Table 4). The aforementioned species had lower abundance, 1–15 specimens, in a minor number of samples (Fig. 2).

**Table 4.** Parameters of the Outlying Mean Index (OMI) for the significant species of Chrysomelidae (p < 0.05) from each season. Values for the non-significant species are presented in Appendix 1: Tables A2, A3. Key: InerO: Total Inertia, T1: Tolerance, T2: Residual tolerance, *p*: probability.

Season	Species	InerO	OMI	T1	T2	*p*
Rainy	*Alagoasatrifasciata* (Fabricius, 1801)	5.199	2.521	0.9	1.778	0.0037
Rainy	*Centralaphthonadiversa* (Baly, 1877)	6.011	0.2003	2.14	3.671	0.0168
Rainy	*Labidomerasuturella* Guérin-Méneville, 1838	23.86	23.86	7.889E-31	-7889-31	0.0409
Rainy	*Monomacrabumeliae* (Schaeffer, 1905)	6.991	0.4444	1.699	4.894	0.0007
Rainy	*Walterianella* sp. 1	7.257	5.25	1.494	0.512	0.0193
Rainy	*Zenocolaspisinconstans* (Lefèvre, 1878)	6.561	4.146	0.233	2.182	0.0172
Dry	*Acallepitrix* sp. 7	8.299	6.09	0.423	1.786	0.0169
Dry	*Alagoasatrifasciata* (Fabricius, 1801)	7.092	6.523	0.038	0.530	0.0469
Dry	*Brachycorynapumila* Guérin-Méneville, 1838	9.761	2.114	5.087	2.56	0.0258
Dry	*Centralaphthonadiversa* (Horn, 1889)	8.056	0.2969	2.788	4.971	0.0415
Dry	*Chaetocnema* sp. 1	10.03	6.778	1.714	1.539	0.0083
Dry	*Epitrix* sp. 1	7.5	3.023	1.432	3.045	0.0073
Dry	*Syphrea* sp. 1	11.29	9.965	0.204	1.12	0.0106

**Figure 3.**
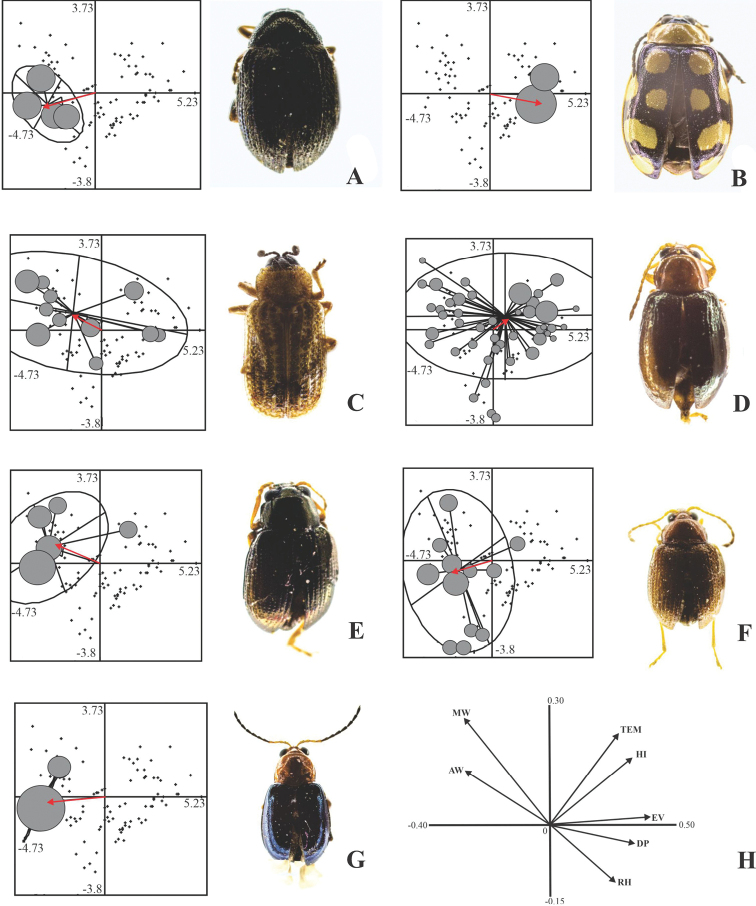
Individual dispersion of leaf beetle species whose association for microclimatic variables was significant in the dry season **A***Acallepitrix* sp. 7 **B***Alagoasatrifasciata***C***Brachycorynapumila***D***Centralaphthonadiversa***E***Chaetocnema* sp. 1 **F***Epitrix* sp. 1 **G***Syphrea* sp. 1. At each species panel: tiny, black dots represent the sampling units; gray circles represent the presence of the species in the sample, and the size of the circle is proportional to its abundance; straight lines represent vectors and indicate the dispersion of the species from the average position (centroid, pointed to by the red arrow) towards each of the sampling units where it was recorded; and ellipses represent the concentration of 95% of the specimens of the species. **H** canonical correlation values ​​(loadings) between microclimatic variables and the abundance of Chrysomelidae. Abbreviations: MW: Maximum wind speed, AW: average wind speed, Tem: temperature, RH: relative humidity, HI: heat index, DP: dew point, Ev: evapotranspiration.

In the case of the dry season, marginality was significant (Monte Carlo test, *p* = 0.017) for only seven of the 40 registered species. Two were considered as generalists, with low marginality values; of these, *B.pumila* presented the highest tolerance, while *C.diversa* showed the lowest marginality (Table 4). Abundance of both species was uniformly distributed in almost all samples (Fig. 3). The other five chrysomelids had high marginality ​​and low tolerance values ​​(specialists): the highest marginality and lowest tolerance occurred in *A.trifasciata*, and it was consequently the species most specialized to microclimatic conditions during the dry season in the semideciduous tropical forest. In descending order, *Syphrea* sp. 1, *Chaetocnema* sp. 1, *Acallepitrix* sp. 7, and *Epitrix* sp. 1 (Table 4) were species recorded in few samples, with abundances ​​between four and 18 specimens (Fig. 3).

Heat index, evapotranspiration and temperature were the microclimatic variables most related with the abundance of leaf beetle species during the rainy season and were represented in Axis 1 of the OMI analysis (Eigenvalue = 4.9077, inertia = 55.74%). In Axis 2 (Eigenvalue = 2.6344, inertia = 29.92%) the most important variable was the average wind speed (Table 5). For the dry season, evapotranspiration, temperature, and heat index in Axis 1 (Eigenvalue = 7.9982, inertia = 75.67%) were the microclimatic variables most associated with the changes in abundance of leaf beetles. Maximum wind speed had the highest correlation in Axis 2 (Eigenvalue = 1.7084, inertia = 0.1616%) (Table 5).

**Table 5.** Canonical correlation values (loadings) between the seven microclimatic variables and the abundance of chrysomelid species during both seasons. Significant values are marked (*).

	Rainy season		Dry season	
Microclimatic variables	Axis 1	Axis 2	Axis 1	Axis 2
Maximum wind speed (m/s)	0.075	0.299	-0.274	0.271*
Average wind speed (m/s)	0.136	0.301*	-0.278	0.143
Temperature (°C)	0.345*	0.042	0.375*	0.192
Relative humidity (%)	-0.084	-0.201	0.2481	-0.119
Heat Index (°C)	0.380*	-0.040	0.371*	0.154
Dew Point (°C)	0.275	-0.173	0.368	-0.001
Evapotranspiration (°C)	0.345*	-0.091	0.413*	0.035

**Figure 4.**
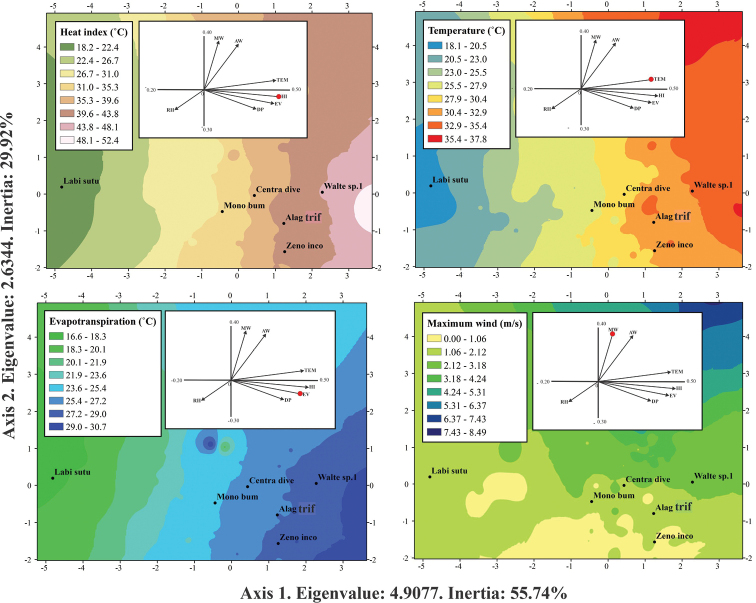
Environmental ranges of leaf beetles during the rainy season. Abbreviations: Labi sutu (*Labidomerasuturella*), Centra dive (*Centralaphthonadiversa*), Mono bume (Monomacrabumeliae), Walte sp. 1 (*Walterianella* sp. 1), Alag trif (*Alagoasatrifasciata*), Zeno inco (*Zenocolaspisinconstans*).

The association of the species with the environmental variables was determined based on the positions of the centroids and their closeness with respect to Axes 1 and 2. Those species that were located very close to the origin of both axes were considered to be related to average microclimatic values. For the rainy season, *A.trifasciata* and *Z.inconstans*, were related with low values ​​of average wind speed (1.06–2.12 m/s), as well as high values ​​of heat index (39.61–43.89 °C), evapotranspiration (27.24–29.02 °C) and temperatures (30.47–35.42 °C). *Walterianella* sp. 1 presented a similar microclimatic pattern, with a positive correlation with Axis 1 (high values of heat index from 43.89 to 48.18 °C, evapotranspiration from 24.24 to 29.02 °C, and temperature from 32.94 to 35.42 °C), although it was associated with average to high values of wind speed (2.12–4.24 m/s). In the case of *L.suturella*, this species was located in areas with lower values of heat index (18.20–22.48 °C), evapotranspiration (16.60–18.37 °C), and temperature (18.10–20.57 °C), but higher wind speed (1.06–2.12 m/s). Lastly, *C.diversa* and *M.bumeliae* did not follow a specific pattern in relation to the significant variables in any axis since they were at the origin of the niche dispersion (Fig. 4).

**Figure 5.**
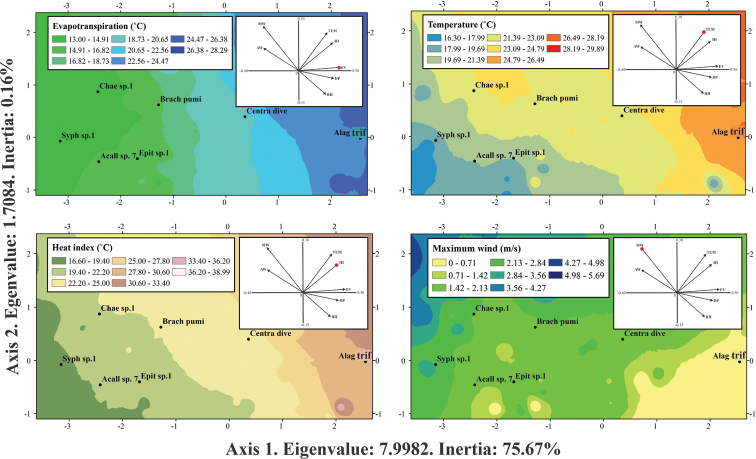
Environmental ranges of leaf beetles during the dry season. Abbreviations: Chae sp. 1 (*Chaetocnema* sp. 1), Syph sp. 1 (*Syphrea* sp. 1), Acall sp. 7 (*Acallepitrix* sp. 7), Brach pumi (*Brachycorynapumila*), Epit sp. 1 (*Epitrix* sp. 1), Centra dive (*Centralaphthonadiversa*), Alag trif (*Alagoasatrifasciata*).

During the dry season, the average distribution of *Syphrea* sp. 1, *Acallepitrix* sp. 7, and *Epitrix* sp. 1 was correlated with areas of lower evapotranspiration (13–16.82 °C), temperature (16.30–19.69 °C) and heat index (16.60–22.20 °C) in Axis 1. Similarly on Axis 2, these species predominated under conditions of low to average maximum wind speed (1.42–2.84 m/s). *Chaetocnema* sp. 1 occurred in conditions of low evapotranspiration (13–14.91 °​​C) and low temperature (21.39–23.09 °C), as well as low heat index (19.40–22.20 °C), but this species was associated with high values ​​of maximum wind speed (1.42–2.13 m/s). *Alagoasatrifasciata* was the species with the lowest tolerance value; so, its centroid was positioned in areas with high evapotranspiration values ​​(22.56–24.47 °C), high temperature (24.79–26.49 °C), high heat index (30.60–33.40 °C), and low maximum wind speed (0–0.71 m/s). Finally, the centroid of the distribution of *B.pumila* and *C.diversa* was significantly associated with average microclimatic conditions, since their distribution included areas with high and low values ​​for the heat index, as well as for the other variables (Fig. 5).

## ﻿Discussion

### ﻿Faunistic inventory and chrysomelid biodiversity

Prior to this study, 2,660 species of Chrysomelidae had been recorded from Mexico (Niño-Maldonado and Sánchez-Reyes 2017) and 257 from the state of Tamaulipas (Niño-Maldonado et al. 2014). Accordingly, results in the STF of the study area represent 2.7% of the leaf beetle biodiversity reported for the country, and 27.6% for the state. Our study revealed *Diachuschlorizans* (Suffrian, 1852) as a new country record for Mexico, and *Diabroticabiannularis* Harold, 1875 as a new state record for Tamaulipas. These records were previously published in preliminary works from the study area (Lucio-Garcia et al. 2019).

The number of taxa recorded in this research is lower compared to similar studies in northeastern Mexico, such as those conducted at El Cielo Biosphere Reserve (RBEC) (Niño-Maldonado et al. 2005), the Cañón de la Peregrina (CDP) (Sánchez-Reyes et al. 2014) and the Sierra de San Carlos (SDSC) (Sánchez-Reyes et al. 2016a). Niño-Maldonado et al. (2005) reported 105 species in different elevational strata of STF. Lower values ​​were found in two fragments of this vegetation in the Peregrina Canyon, where 85 (Sánchez-Reyes et al. 2014) and 37 species (Martínez-Sánchez 2016) were recorded. Other patches of STF were evaluated in the Cañón del Novillo (21 species) and Cerro El Diente (five species), also in Tamaulipas. The lower number in the present study can be attributed to the spatial scale and number of environments evaluated in these other investigations, which are greater compared to the STF of this work. For example, analyzing elevation gradients, different types of vegetation or biogeographic islands with extreme conservation status may result in the observed differences in fauna. On basis of the aforementioned numbers, the chrysomelid richness for the current research is above other STF fragments in northeastern Mexico, and it represents 68.2% of the most biodiverse site. Regarding true diversity, the numbers of equally dominant (1/D) and typical (e^H^) species in this study were lower than those observed in Peregrina Canyon (Sánchez-Reyes et al. 2014), although they were higher than those observed in STF fragments from Cañón del Novillo (Martínez-Sánchez 2016) or Cerro El Diente (Sánchez-Reyes et al. 2015b).

Galerucine dominance as observed in our study has also been reported in other studies in northeastern Mexico (Niño-Maldonado et al. 2005; Furth 2009; Furth 2013; Sánchez-Reyes et al. 2013; Sánchez-Reyes et al. 2014; Bouzan et al. 2015; Flinte et al. 2017; Lucio-García et al. 2019, 2020), and this may be due to the subfamily’s high number of species (Riley et al. 2002), with specimens found in all ecosystems during most parts of the year (Furth 2013; Sánchez-Reyes et al. 2013). In contrast, the subfamily composition of this study is quite different from that in tropical forests. In the Chamela region, on the Pacific side of Mexico, 49 species of Cassidinae were listed (Noguera 1988).

As a whole, the aforementioned results highlight the great importance of the study area, since it was possible to find a large percentage of species within a smaller expanse when compared to larger space-temporal gradients or natural protected areas. This can be attributed to the geographic location of the studied STF within a region with a high conservation priority (CONABIO et al. 2007). The area, although adjacent to the Altas Cumbres Natural Protected Area, constitutes a mosaic with fragments of different durations since last disturbance, and this may favor the presence of a complex community of species (Sánchez-Reyes et al. 2017). Furthermore, the STF is one of the ecosystems with the highest biodiversity of plants (Rzedowski 2006; García-Morales et al. 2014), and it is one of the most important in terms of chrysomelid species richness in Mexico (Noguera 1988; Burgos-Solorio and Anaya-Rosales 2004; Niño-Maldonado et al. 2005; Lucio-García et al. 2019). The combination of environmental factors in the STF results in a great diversification of plants, providing a wide range of food resources, which could lead to the high number of leaf beetles in this plant community in Tamaulipas and other states of Mexico.

### ﻿Seasonal variation

On a temporal scale, the chrysomelid community followed a seasonal pattern, where the rainy season was the most favorable for the presence of this group in the study area. Increase in abundance and species richness during this season has also been found in numerous studies worldwide, including studies in Tamaulipas and other parts of Mexico (Petitpierre et al. 2000; Esker et al. 2002; Burgos-Solorio and Anaya-Rosales 2004; Koji and Nakamura 2006; Furth 2009; Martínez-Sánchez et al. 2009; Furth 2013; Sánchez-Reyes et al. 2015b; Sánchez-Reyes et al. 2016a; Sandoval-Becerra et al. 2016; Şen and Gök 2016; Miwa and Meinke 2017; Lucio-García et al. 2019). Results of the richness estimators support these patterns, because the percentage of completeness during rains is lower when compared to the dry season. Thus, in certain areas, the highest activity of chrysomelids is restricted to the rainy season, while inactivity increases during drought conditions (Noguera 1988; Furth 2013). This is due to the association of chrysomelids with the quality and availability of their host plants (Řehounek 2002; Şen and Gök 2016), which are some of the most important elements in their diet (Ávila and Postali-Parra 2003), as well as with the abundance of young foliage (Basset and Samuelson 1996), variables that are increased during the period of highest rainfall. In addition, there is more vegetation cover producing shade, creating microenvironments that could be more favorable to maintaining a high population density (Hill and Hill 2001), with the climatic conditions of humidity necessary for the adult beetles to emerge and fly (Yanes-Gómez and Morón 2010).

However, in other geographic regions, such as the subtropical areas of Brazil, the highest abundance has occurred in the dry season, specifically within the subfamilies Galerucinae, Cassidinae and Chrysomelinae (Linzmeier and Ribeiro-Costa 2008; Flinte et al. 2011; Bouzan et al. 2015; Flinte et al. 2017). In addition, in some areas of northeastern Mexico, greater numbers of species and specimens have also been recorded during the dry season (Sánchez-Reyes et al. 2014). These discrepancies can be attributed to the climatic and biogeographic differences between plant communities. For example, in cloud forests, dry periods are shorter and less intense, causing a favorable increase in specimens of some Coleoptera families (Pedraza et al. 2010). Although soil moisture and precipitation are reduced in these areas, the cloudiness in the form of mist reduces evaporation, providing water during periods of low rain; in consequence, marked deficiency of humidity in these forests is rare. In other tropical forests near the study area, the dry season is not as severe, for example in the Peregrina Canyon, where a higher abundance of adult chrysomelids often occurs concentrated in refuges during this season, while the larval stages are more abundant during the rains (Sánchez-Reyes et al. 2014). On the contrary, differences in geographic position, latitude and elevation influence the contrast that exists between the dry and rainy seasons in other fragments of the same type of vegetation in northeastern Mexico. In the study area, there are well-defined periods of high temperature and precipitation, in addition to a non-continuous flow of water currents during the year, which lead to a more severe dry season. Similar and more extreme cases exist in tropical dry forests from the south or Pacific coast of Mexico, where the plants lose their leaves completely during the dry season, resulting in a notable absence of chrysomelids (Noguera 1988). Likewise, these climatic variations and their effects on the phenology of the host plants are probably the main drivers of the temporal dynamics in these beetles (Flinte et al. 2017).

Unlike other investigations where the greatest diversity also occurs in the wet season (Sánchez-Reyes et al. 2016a), in this work, the low abundance and species richness resulted in a high diversity in the dry season, by decreasing the dominance and increasing the effective number of species (Magurran 2004). Therefore, the dry season is of great importance for the chrysomelid community in the STF of the study area, since the prevailing conditions increase the evenness of the chrysomelid community. Species may exploit food resources in a more efficient way during this season, achieving a balance in their populations and reducing the dominance of most species, thus suggesting an adaptation of Chrysomelidae to acute drought conditions. This could be noted also when observing the high percentage of faunistic similarity between seasons, which indicates that most of the leaf beetles are the same in both periods. Therefore, it is possible that their resource acquirement changes and consequently their abundances are modified during the seasonal variations. Moreover, 31 species were registered exclusively for the rainy season, while only 11 for the dry season. Together, these results highlight the relevance of areas where there is a marked temporal or seasonal heterogeneity, since it can generate unique species compositions.

### ﻿Response of Chrysomelidae to seasonal microclimatic changes

In this research, the niches of chrysomelid species were examined by means of the Outlying Mean Index. This showed that the variations in the abundance of leaf beetles were significantly related to the microclimatic changes in each season. Factors that influence the distribution of phytophagous insects are a combination of geographic and environmental elements (Wąsowska 2004; Andrew and Hughes 2005; Lassau et al. 2005; Baselga and Jiménez-Valverde 2007). It has also been shown that leaf beetles present different degrees of association with the microclimatic conditions of the habitats where they develop (Sánchez-Reyes et al. 2016b; Sandoval-Becerra et al. 2017), and this is demonstrated in our study. However, the variation explained by the analysis and the correlation values ​​of the variables were higher in the dry season, suggesting a stronger association between the microclimate and the chrysomelid community with respect to the rainy season. This can be attributed to more heterogeneous environment values during low precipitation months. For example, in tropical forests it has been observed that lower microclimatic variability occurs through the rainy season (Checa et al. 2014; Sánchez-Reyes et al. 2019), which could be due to a higher homogeneity in the vegetation structure. Therefore, chrysomelid populations are more variable in relation to the seasonal microclimate prevailing during the dry season, so that the effects, particularly of precipitation, determine strong positive or negative responses in these insects (Pinheiro et al. 2002); this pattern also occurs in other phytophagous groups, such as Curculionidae or Cicadidae (Novotny et al. 1999; Silva et al. 2017).

Significant microclimatic variables were very similar between seasons (environmental temperature, heat index, evapotranspiration and, to a lesser extent, wind speed), although there were differences in the order of importance and in their contribution to the variations in abundance of leaf beetles. In the rainy season, the most important variable to characterize the niche of the species was the heat index, which is considered to be a combination of humidity and temperature in the same value and represents the thermal sensation (Lee and Brenner 2015). In physiological terms, phytophagous insects must accumulate a certain amount of heat to be able to hatch and accelerate their development rate, thereby increasing the number of generations (Marco 2001; Mejía 2005). However, in the dry season, the variable of greatest importance was evapotranspiration. Such variation can be attributed to the environmental humidity stress to which the host plants are exposed after rainfall, modifying the moisture content of leaves and stems and thereby affecting feeding patterns of chrysomelids. This suggests that direct effects on trophic networks may occur during drought periods, which influence the development of phytophagous insects, particularly due to desiccation (Martínez et al. 2010; Anderson et al. 2016).

A similar set of microclimatic variables has been associated with Chrysomelidae in other works, specifically temperature, heat index, maximum wind speed and evapotranspiration (Stewart et al. 1996; Flinte and Valverde de Macedo 2004; Isard et al. 2004; Baselga and Jiménez-Valverde 2007; Linzmeier and Ribeiro-Costa 2013; Aneni et al. 2014; Sánchez-Reyes et al. 2016b; Oliveira et al. 2017; Sandoval-Becerra et al. 2017). There are other studies where the most important abiotic variables were solar radiation, precipitation, relative humidity, photoperiod and condensation point (Flinte and Valverde de Macedo 2004; Isard et al. 2004; Linzmeier and Ribeiro-Costa 2013). It should be mentioned that differences compared to the present study arise due to various factors, including the type of study, geographic location, ecosystems evaluated and method for measuring microclimatic variables, as well as the specific response of taxa to the variables.

Regarding the individual response of leaf beetles to the variables, it was observed that only 11 of the 71 species registered a significant variation between their niche and the average microclimatic conditions in the STF. It has been observed that the number of chrysomelid species that present a significant relationship with abiotic parameters is variable, although previous studies have focused on the effect of disturbance (Sandoval-Becerra et al. 2017) and elevation gradients (Sánchez-Reyes et al. 2016b). The study of chrysomelid species associated with abiotic variables has been useful in recognizing part of their biology and ecology, specifically their reproductive cycle or their potential for biological control (Stewart et al. 1996; Flinte and Valverde de Macedo 2004; Isard et al. 2004; Oliveira et al. 2017). Also, such study has been applied to know their niches (Sánchez-Reyes et al. 2015a) and to identify indicator species of conservation status (Ohsawa and Nagaike 2006) or disturbance (Sandoval-Becerra et al. 2017). Other studies have focused on the influence of climate in the distribution of species (Sánchez-Reyes et al. 2016b; Wang et al. 2017). The present work, on the other hand, is one of the first to address the influence of microclimatic variation on Chrysomelidae from a seasonal perspective.

Specifically, in the dry season, seven significant species were recorded, while in the rainy season there were only six. In both seasons, *C.diversa* was categorized as a generalist species, since it presented a low marginality and a high tolerance, which indicates a wide distribution in the study area associated with average microclimatic values. This response is similar to that observed in the same and other species within the genus, but in different areas (Sánchez-Reyes et al. 2016b; Sandoval-Becerra et al. 2017). The second common species in both seasons was *A.trifasciata* although it was categorized as a specialist due to high marginality and low tolerance values. A similar response pattern was previously recorded in the Sierra de San Carlos for this species (Sánchez-Reyes 2014). Association between variables and *A.trifasciata* was higher in the dry season, since the marginality parameters were higher, while the tolerance values ​​were lower; that is, the distribution of *A.trifasciata* appears to be more restricted during the dry conditions. The rest of the species demonstrated seasonal differences. For example, during the rainy season, *L.suturella* presented the highest marginality value and had a low tolerance to the microclimatic environment; similar responses were observed in *Walterianella* sp. 1 and *Z.inconstans*. In the dry season, *Syphrea* sp. 1 was the species with the lowest tolerance, followed by *Chaetocnema* sp. 1, *Acallepitrix* sp. 7, *Epitrix* sp. 1 (Galerucinae), and *B.pumila* (Cassidinae). However, some of these species also occurred throughout the year, despite being significantly associated with only one season. The above observations provide evidence that leaf beetles have seasonal modifications in their niche requirements. Influence of the microclimate may be more important in the rainy season, while in the dry season (or vice versa) the variables that determine niches are different, or they may have a non-significant contribution to the distribution of the species (Basset et al. 1992; Martínez et al. 2010; García-Atencia et al. 2015). These seasonal changes may be associated with the synchronization of the reproductive cycles of the phytophagous insects, particularly depending on the precipitation and temperature provided by the forest structure, which is not constant throughout the year and tends to be increasingly variable (Basset et al. 1992; Soler et al. 2002; García-Atencia et al. 2015).

The broad microclimatic tolerance of *C.diversa* and the abiotic specialization of *A.trifasciata* represent a first approach to the analysis of the generalized environmental response of chrysomelids, even though both have been documented in other studies. In this way, it is probable that the behavior of the species is similar and constant in other geographical areas, which would allow the use of such taxa in environmental monitoring. New studies on chrysomelid niches would allow us to elucidate these effects. It is also important to recognize that phytophagous insects and specialist taxa with a small niche breadth could be negatively influenced by the possible effects of climate change (Williams et al. 2007; Dormann et al. 2008; Hill et al. 2011), which will impact the structure and functioning of the communities (Hegland et al. 2009; Stuble et al. 2013; Luna-Castellanos et al. 2017). Effects extend to plant-insect interactions (mutualism, predation, competition, etc.), either due to phenological changes (synchronization in the interaction) or distribution of species (Hódar et al. 2004; Luna-Castellanos et al. 2017), with some species even being susceptible to local extinction (Tscharntke et al. 2002; Petermann et al. 2010). Furthermore, the present results and similar evidence suggest that climate variability can lead to significant biodiversity losses (Parmesan et al. 1999; Hill et al. 2002; Konvicka et al. 2003; Wilson et al. 2007). However, despite having knowledge about possible consequences, little information is available on the effects that the changing microclimate can have on biodiversity, its populations, biological communities, and the ecosystems that harbor them.

## ﻿Conclusions

The study of seasonal and microclimatic changes on species and communities is a topic of great importance in conservation ecology. Community attributes of the family Chrysomelidae and the beetles’ response to microclimatic variation were evaluated for the first time from a seasonal perspective, in a semideciduous tropical forest fragment of northeastern Mexico. Overall, the observed results were similar to those from other faunistic studies of leaf beetles, although the number of species ranked third within tropical forest areas of the state of Tamaulipas. Seasonality induced significant changes in the parameters of abundance, diversity and faunistic composition in the chrysomelid community. The highest number of specimens and species were recorded in the rainy season, while the lowest dominance and highest diversity occurred in the driest period.

In this study, it was shown that Chrysomelidae were significantly associated with the microclimatic variation among seasons. However, the strength of this association and the number of significant species were different for each season. Changes in the abundance of the leaf beetles were influenced by the heat index, temperature, evapotranspiration, and average wind speed, reflected by specific conditions required for each species. Microclimatic and seasonal assessment could be useful for the evaluation of climate change, since niche analysis enables detection of specialized or vulnerable species, which are associated with a delimited set of environmental conditions. This characterization of the microclimate niche of Chrysomelidae from a seasonal perspective was conducted here for the first time in northeastern Mexico. However, additional studies are warranted to determine if the observed patterns are different when evaluating other abiotic factors or when evaluating other plant communities.

## ﻿Appendix 1

**Table A1.** Taxonomic checklist of Chrysomelidae by season in a fragment of semideciduous tropical forest from northeastern Mexico (March 2016 to February 2017).

Taxon		Rainy season	Dry season
	N	N	N
**CRIOCERINAE Latreille, 1807**	**14**		
Tribe Lemini Heinze, 1962			
*Lema* sp. 1	5	–	5
*Lema* sp. 2	2	2	–
*Lema* sp. 3	2	–	2
*Neolema* sp. 1	2	2	–
*Oulema* sp. 1	3	3	–
**CASSIDINAE Gyllenhal, 1813**	**410**		
Tribe Chalepini Weise, 1910			
*Baliosus* sp. 1	1	1	–
*Brachycorynapumila* Guérin-Méneville, 1844	34	17	17
*Chalepusdigressus* Baly, 1885	1	–	1
*Heterispavinula* (Erichson, 1847)	311	209	102
*Octotomaintermedia* Staines, 1989	3	3	–
*Sumitrosisinaequalis* (Weber, 1801)	2	2	–
Tribe Cassidini Gyllenhal, 1813			
*Agroiconotavilis* (Boheman, 1855)	1	1	–
*Charidotellasexpunctata* (Fabricius, 1781)	3	2	1
*Helocassisclavata* (Fabricius, 1798)	12	5	7
*Helocassiscrucipennis* (Boheman, 1855)	37	16	21
*Microctenochirapunicea* (Boheman, 1855)	4	3	1
*Microctenochiravaricornis* (Spaeth, 1926)	1	1	–
**CHRYSOMELINAE Latreille, 1802**	**9**		
Tribe Chrysomelini Latreille, 1802			
*Calligraphaancoralis* Stål, 1860	1	1	–
*Calligraphafulvipes* Stål, 1859	1	–	1
*Deuterocamptaatromaculata* Stål, 1859	1	1	–
*Labidomerasuturella* Chevrolat, 1844	3	1	2
*Plagioderasemivittata* Stål, 1860	2	1	1
*Plagioderathymaloides* Stål, 1860	1	1	–
**GALERUCINAE Latreille, 1802**	**1628**		
Tribe Galerucini Latreille, 1802			
*Coraiasubcyanescens* (Schaeffer, 1906)	8	8	–
Tribe Luperini Chapuis, 1875			
*Acalymma* sp. 1	1	1	–
*Cyclotrypemafurcata* (Olivier, 1808)	23	23	–
*Diabroticabiannularis* Harold, 1875	1	1	–
*Gynandrobroticalepida* (Say, 1835)	8	1	7
*Paratriariuscurtisii* (Baly, 1886)	1	1	–
Tribe Alticini Newman, 1835			
*Acallepitrix* sp. 1	1	–	1
*Acallepitrix* sp. 2	1	1	–
*Acallepitrix* sp. 3	2	2	–
*Acallepitrix* sp. 4	3	–	3
*Acallepitrix* sp. 5	11	5	6
*Acallepitrix* sp. 6	9	2	7
*Acallepitrix* sp. 7	8	4	4
*Acallepitrix* sp. 8	2	2	–
*Acrocyumdorsale* Jacoby, 1885	30	17	13
*Acrocyum* sp. 1	2	–	2
*Alagoasabipunctata* (Chevrolat, 1834)	8	5	3
*Alagoasatrifasciata* (Fabricius, 1801)	19	15	4
*Alagoasa* sp. 1	1	1	–
*Asphaeraabdominalis* (Chevrolat, 1835)	1	1	–
*Asphaeranigrofasciata* Jacoby, 1885	1	1	–
*Centralaphthonadiversa* (Baly, 1877)	692	440	252
*Centralaphthona* sp. 1	1	1	–
*Chaetocnema* sp. 1	19	6	13
*Disonychastenosticha* Schaefer, 1931	1	–	1
*Epitrix* sp. 1	28	10	18
*Heikertingerella* sp. 1	24	21	3
*Longitarsus* sp. 1	7	4	3
*Longitarsus* sp. 2	16	1	15
*Margaridisa* sp. 1	147	16	131
*Monomacrabumeliae* (Schaeffer, 1905)	528	336	192
*Phyllotretaaeneicollis* (Crotch, 1873)	1	1	–
*Syphrea* sp. 1	8	2	6
*Syphrea* sp. 2	5	5	–
*Walterianella* sp. 1	9	8	1
*Walterianella* sp. 2	1	1	–
**CRYPTOCEPHALINAE Gyllenhal, 1813**	**6**		
Tribe Cryptocephalini Gyllenhal, 1813			
*Cryptocephalusumbonatus* Schaeffer, 1906	1	–	1
*Diachuschlorizans* (Suffrian, 1852)	1	1	–
Tribe Clytrini Lacordaire, 1848			
*Babiadistinguenda* Jacoby, 1889	1	1	–
*Smaragdinaagilis* (Lacordaire, 1848)	3	–	3
**EUMOLPINAE Hope, 1840**	**36**		
Tribe Eumolpini Hope, 1840			
*Brachypnoea* sp. 1	3	1	2
*Brachypnoea* sp. 2	5	1	4
*Colaspisfreyi* (Bechyné, 1950)	1	1	–
*Colaspismelancholica* Jacoby, 1881	13	12	1
*Colaspistownsendi* Bowditch, 1921	1	1	–
*Xanthonia* sp. 1	3	–	3
*Zenocolaspisinconstans* (Lefèvre, 1878)	8	7	1
Tribe Typophorini Chapuis, 1874			
*Paria* sp. 1	2	2	–
**71 species Totals**	**2103**	**1242**	**861**

**Table A2.** Outlying Mean Index parameters for chrysomelid species in the rainy season. Key: InerO = Total inertia, OMI = Marginality index, T1 = Tolerance, T2 = Residual tolerance, *p* = probability; significant values in bold.

Species	InerO	OMI	T1	T2	P
*Acallepitrix* sp. 2	3.76	3.76	0.00	0.00	0.55
*Acallepitrix* sp. 3	11.91	5.64	3.86	2.42	0.16
*Acallepitrix* sp. 5	5.74	2.92	0.82	2.00	0.09
*Acallepitrix* sp. 6	11.66	7.62	2.35	1.69	0.08
*Acallepitrix* sp. 7	3.88	0.23	0.99	2.67	0.96
*Acallepitrix* sp. 8	3.72	3.03	0.10	0.59	0.42
*Acalymma* sp. 1	4.56	4.56	0.00	0.00	0.47
*Acrocyumdorsale*	6.33	0.54	2.01	3.78	0.24
*Agroiconotavilis*	1.51	1.51	0.00	0.00	0.91
*Alagoasabipunctata*	3.45	0.85	0.97	1.63	0.70
***Alagoasatrifasciata***	**5.20**	**2.52**	**0.90**	**1.78**	**0.00**
*Alagoasa* sp. 1	2.29	2.29	0.00	0.00	0.78
*Asphaeraabdominalis*	2.29	2.29	0.00	0.00	0.78
*Asphaeranigrofasciata*	5.57	5.57	0.00	0.00	0.40
*Babiadistinguenda*	9.82	9.82	0.00	0.00	0.23
*Brachycorynapumila*	5.14	0.95	2.58	1.62	0.25
*Brachypnoea* sp. 1	5.83	5.83	0.00	0.00	0.37
*Brachypnoea* sp. 2	0.75	0.75	0.00	0.00	0.97
*Calligraphafulvipes*	3.05	3.05	0.00	0.00	0.65
***Centralaphthonadiversa***	**6.01**	**0.20**	**2.14**	**3.67**	**0.02**
*Centralaphthona* sp. 1	0.94	0.94	0.00	0.00	0.96
*Chaetocnema* sp. 1	5.46	1.58	0.81	3.08	0.43
*Charidotellasexpunctata*	2.52	1.27	0.62	0.63	0.79
*Colaspisfreyi*	4.30	4.30	0.00	0.00	0.49
*Colaspismelancholica*	7.96	7.96	0.00	0.00	0.31
*Colaspistownsendi*	3.33	1.00	0.24	2.08	0.40
*Coraiasubcyanescens*	4.61	0.57	0.42	3.62	0.78
*Cyclotrypemafurcata*	3.25	0.37	0.70	2.18	0.58
*Diabroticabiannularis*	2.22	2.22	0.00	0.00	0.81
*Diachuschlorizans*	0.59	0.59	0.00	0.00	0.98
*Deuterocamptaatromaculata*	3.05	3.05	0.00	0.00	0.65
*Epitrix* sp. 1	4.62	3.55	0.37	0.70	0.07
*Gynandrobroticalepida*	20.23	20.23	0.00	0.00	0.06
*Heikertingerella* sp. 1	6.29	0.11	1.29	4.89	0.96
*Helocassisclavata*	9.99	1.93	6.06	2.00	0.22
*Helocassiscrucipennis*	5.12	1.61	0.46	3.05	0.09
*Heterispavinula*	6.06	0.09	1.30	4.68	0.21
***Labidomerasuturella***	**23.86**	**23.86**	**1.83**	**0.00**	**0.04**
*Lema* sp. 2	6.01	4.36	0.80	0.85	0.26
*Longitarsus* sp. 1	17.20	5.69	10.66	0.85	0.07
*Longitarsus* sp. 2	2.53	2.53	0.00	0.00	0.75
*Margaridisa* sp. 1	5.40	0.16	2.07	3.17	0.86
*Microctenochirapunicea*	2.59	1.41	0.38	0.80	0.59
*Microctenochiravaricornis*	9.80	9.80	0.00	0.00	0.23
***Monomacrabumeliae***	**6.99**	**0.44**	**1.70**	**4.85**	**0.00**
*Neolema* sp. 1	5.55	5.45	0.00	0.10	0.18
*Octotoma* sp. 1	4.60	1.23	1.88	1.50	0.66
*Oulema* sp. 1	3.75	2.72	0.08	0.94	0.49
*Paratriariuscurtisii*	2.62	2.62	0.00	0.00	0.71
*Paria* sp. 1	8.16	2.32	3.98	1.86	0.56
*Phyllotretaaeneicollis*	6.34	6.34	0.00	0.00	0.35
*Plagioderasemivittata*	4.00	4.00	0.00	0.00	0.53
*Plagioderathymaloides*	3.15	3.15	0.00	0.00	0.65
*Sumitrosisinaequalis*	3.17	0.35	0.64	2.18	0.97
*Sumitrosis* sp. 1	1.24	1.24	0.00	0.00	0.94
*Syphrea* sp. 1	1.70	0.60	0.01	1.08	0.92
*Syphrea* sp. 2	3.41	3.20	0.04	0.16	0.44
***Walterianella* sp. 1**	**7.26**	**5.25**	**1.49**	**0.51**	**0.02**
*Walterianella* sp. 2	2.62	2.62	0.00	0.00	0.71
***Zenocolaspisinconstans***	**6.56**	**4.15**	**0.23**	**2.18**	**0.02**

**Table A3.** Outlying Mean Index parameters for chrysomelid species in the dry season. Key: InerO = Total inertia, OMI = Marginality index, T1 = Tolerance, T2 = Residual tolerance, *p* = probability; significant values in bold.

Species	InerO	OMI	T1	T2	P
*Acallepitrix* sp. 1	7.86	7.86	0.00	0.00	0.41
*Acallepitrix* sp. 4	5.76	2.15	0.23	3.38	0.41
*Acallepitrix* sp. 5	5.63	0.90	3.16	1.58	0.45
*Acallepitrix* sp. 6	6.31	0.46	2.41	3.43	0.80
***Acallepitrix* sp. 7**	**8.30**	**6.09**	**0.42**	**1.79**	**0.02**
*Acrocyumdorsale*	8.21	0.25	3.58	4.38	0.72
*Acrocyum* sp. 1	14.37	7.55	4.42	2.40	0.10
***Alagoasatrifasciata***	**7.09**	**6.52**	**0.04**	**0.53**	**0.05**
*Alagoasa* sp. 1	4.47	1.72	0.68	2.07	0.52
***Brachycorynapumila***	**9.76**	**2.11**	**5.09**	**2.56**	**0.03**
*Brachypnoea* sp. 1	9.51	0.65	4.61	4.25	0.95
*Brachypnoea* sp. 2	12.33	12.33	0.00	0.00	0.10
*Calligraphafulvipes*	5.45	5.45	0.00	0.00	0.56
***Centralaphthonadiversa***	**8.06**	**0.30**	**2.79**	**4.97**	**0.04**
***Chaetocnema*** sp. 1	**10.03**	**6.78**	**1.71**	**1.54**	**0.01**
*Chalepusdigressus*	12.80	12.80	0.00	0.00	0.09
***Charidotellasexpunctata***	9.42	9.42	0.00	0.00	0.22
*Colaspistownsendi*	5.85	5.85	0.00	0.00	0.52
*Cryptocephalusumbonatus*	0.83	0.83	0.00	0.00	0.98
*Disonychastenosticha*	8.31	8.31	0.00	0.00	0.32
***Epitrix* sp. 1**	**7.50**	**3.02**	**1.43**	**3.05**	**0.01**
*Gynandrobroticalepida*	4.94	1.29	0.25	3.41	0.24
*Heikertingerella* sp. 1	5.75	0.88	2.31	2.57	0.76
*Helocassisclavata*	6.28	0.57	4.26	1.45	0.58
*Helocassiscrucipennis*	8.65	1.95	5.37	1.33	0.13
*Heterispavinula*	7.04	0.23	1.79	5.02	0.15
*Labidomerasuturella*	3.43	1.30	0.78	1.34	0.82
*Lema* sp. 1	8.29	1.54	4.25	2.50	0.82
*Lema* sp. 3	2.29	2.29	0.00	0.00	0.89
*Longitarsus* sp. 1	5.51	2.46	0.09	2.96	0.34
*Longitarsus* sp. 2	5.79	0.13	0.20	5.47	0.88
*Margaridisa* sp. 1	7.69	0.13	3.27	4.29	0.96
*Microctenochirapunicea*	2.93	2.93	0.00	0.00	0.76
*Monomacrabumeliae*	6.20	0.16	2.86	3.18	0.08
*Plagioderathymaloides*	5.72	5.72	0.00	0.00	0.54
*Smaragdinaagilis*	5.31	0.53	1.71	3.07	0.97
***Syphrea* sp. 1**	**11.29**	**9.97**	**0.20**	**1.12**	**0.01**
*Walterianella* sp. 1	5.72	5.72	0.00	0.00	0.54
*Xanthonia* sp. 1	4.60	2.42	0.23	1.95	0.36
*Zenocolaspisinconstans*	4.25	4.25	0.00	0.00	0.69
